# Maintenance of Effectiveness of Pirfenidone in Elderly Patients with Progressive Functional Impairment: A Real-World Retrospective Study in IPF †

**DOI:** 10.3390/biomedicines13112809

**Published:** 2025-11-18

**Authors:** Stefano Levra, Cecilia Rivero, Fabiana Giannoccaro, Giuseppe Guida, Francesca Bertolini, Vitina Carriero, Elisa Arrigo, Maurizio Balbi, Carlo Albera, Fabio Luigi Massimo Ricciardolo

**Affiliations:** 1 Department of Clinical and Biological Sciences, University of Turin, Regione Gonzole 10, 10043 Orbassano, Italy; cecilia.rivero@unito.it (C.R.); giuseppe.guida@unito.it (G.G.); francesca.bertolini@unito.it (F.B.); vitina.carriero@unito.it (V.C.); elisa.arrigo@unito.it (E.A.); fabioluigimassimo.ricciardolo@unito.it (F.L.M.R.); 2 Severe Asthma, Rare Lung Disease and Respiratory Pathophysiology Unit, San Luigi Gonzaga University Hospital, Regione Gonzole 10, 10043 Orbassano, Italy; f.giannoccaro@sanluigi.piemonte.it; 3 Radiology Unit, Department of Oncology, San Luigi Gonzaga University Hospital, Regione Gonzole 10, 10043 Orbassano, Italy; maurizio.balbi@unito.it; 4 Department of Medical Sciences, University of Turin, Corso Achille Mario Dogliotti 14, 10126 Turin, Italy; carlo.albera@yahoo.it; 5 Division of Respiratory Medicine, Cardiovascular and Thoracic Department, AOU Città della Salute e della Scienza di Torino, Corso Bramante 88, 10126 Turin, Italy

**Keywords:** idiopathic pulmonary fibrosis, pirfenidone, effectiveness, age, advanced disease

## Abstract

**Background/Objectives:** In clinical trials designed to evaluate efficacy and safety of pirfenidone in patients with idiopathic pulmonary fibrosis (IPF), inclusion criteria were age ≤ 80 years, FVC ≥ 50%pred and DLco ≥ 35%pred. The outcomes in patients progressing beyond these criteria are not yet fully investigated. This study aims to evaluate the effectiveness of pirfenidone in patients who, during treatment, progress beyond the criteria adopted in clinical trials. **Methods**: This observational retrospective single-centre study included patients younger than 81 years and with mild-to-moderate IPF who had initiated pirfenidone from December 2011 to October 2023. We compared the monthly decline in absolute FVC and %DLco before and after the progression beyond one or more inclusion criteria used in clinical trials. **Results**: A total of 174 patients were included in the study, with a mean follow-up of 39.2 months (SD ± 29.7 months, range 2–152 months). Seventy-six of them remained within all criteria (control group), 72 passed one criterion, 25 two criteria and 1 all criteria. There was no difference in the trend of FVC and %DLco between the control group and the groups that passed one or two criteria. The intra-individual trend after passing one criterion was similar to that shown before, for both FVC and %DLco. The intra-individual trend also appeared to be preserved when analyzing a small group of patients who had passed any two criteria. **Conclusions**: Neither attaining advanced age nor the development of severe functional impairment appeared to limit the effectiveness of pirfenidone.

## 1. Introduction

Pirfenidone was the first antifibrotic drug approved for the treatment of idiopathic pulmonary fibrosis (IPF) [1]. The pivotal clinical trials were published in 2011 and included patients with age ≤80 years and mild-to-moderate disease, defined as a percentage predicted forced vital capacity (%FVC) ≥50% and a percentage predicted diffusion capacity of the lung for carbon monoxide (%DLco) ≥35% [2,3].

In the following years, some post hoc analysis of phase 3 studies investigated the safety and efficacy of the drug in patients with more advanced disease, most often defined as %FVC < 50% and/or %DLco < 35%. In particular, a post hoc analysis including patients with advanced IPF in the ASCEND (NCT01366209) and CAPACITY (NCT00287716 and NCT00287729) trials showed that pirfenidone can lead to several clinically significant benefits across multiple areas even in this population, without an increased risk of discontinuation due to adverse events [4]. Post hoc analysis of the extension study RECAP (NCT00662038) also confirmed that long-term pirfenidone treatment results in a similar decline in lung function in patients with more advanced than those with less advanced IPF [5]. Therefore, the indication in the European Union has recently been expanded by the European Medicines Agency to include the treatment of patients with advanced IPF, ensuring an effective treatment also for this relevant group of patients [6].

However, several issues remain to be further clarified. In particular, most studies have focused on comparing patients with advanced IPF to patients with mild-to-moderate disease, but little is known about effectiveness in those who develop an advanced form of the disease during treatment. Similarly, effectiveness in patients with advanced impairment of both %FVC and %DLco remains to be better investigated, as well as in patients older than 80 years. Nevertheless, in daily practice, treatment is usually continued regardless of age or the development of advanced disease.

Considering the lack of data in the literature, the aim of this study was to evaluate the effectiveness of pirfenidone in patients who, during follow-up, had progressed beyond one or more inclusion criteria used in pivotal clinical trials.

This article is a revised and expanded version of a conference paper entitled “Effectiveness of pirfenidone in elderly patients with advanced functional impairment: a real-world study in idiopathic pulmonary fibrosis”, which was presented as an abstract at the 2024 European Respiratory Society Congress [7].

## 2. Materials and Methods

### 2.1. Study Design and Participants

This is an observational retrospective single-centre study. Data were collected from the medical records available at the “Rare Lung Disease Centre” of the San Luigi Gonzaga University Hospital (Orbassano, Turin, Italy). This study was conducted according to the principles of the Declaration of Helsinki and approved by the local Ethics Committee (protocol No. 239/2021). Written informed consent was obtained from the patients.

This study included all patients younger than 81 years and with mild-to-moderate IPF who had started pirfenidone between December 2011 and October 2023. The diagnosis had been confirmed according to the joint consensus reports of the American Thoracic Society (ATS), European Respiratory Society (ERS), Japanese Respiratory Society (JRS), and Latin American Thoracic Association (ALAT) [8,9,10]. We collected respiratory function data of these patients from the start of treatment to the end of January 2024. Collection was interrupted in case of drug discontinuation, lung transplantation, or death.

**Figure 1:**
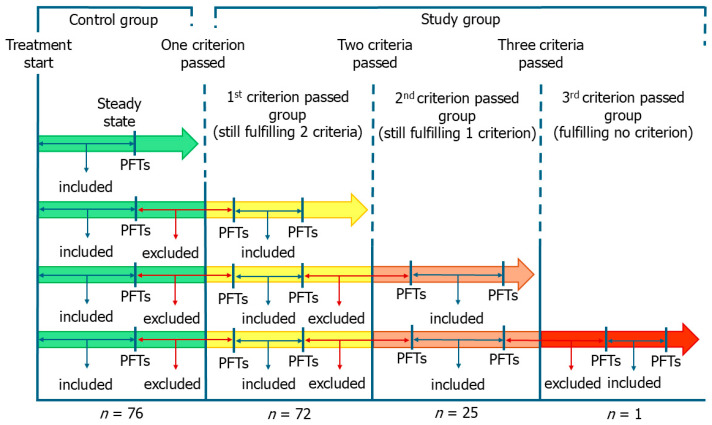
Study design and group division. Patients were categorized based on the occurrence of functional deterioration or ageing events in the period following the start of pirfenidone, regardless of the time interval since the start. Patients who remained within the inclusion criteria used in the clinical trials (steady state) throughout follow-up formed the control group. All the others formed the study group, which was divided into three subgroups according to the number of criteria passed. We excluded from the analysis the respiratory function data corresponding to the months in which a criterion was passed. PFTs, pulmonary function tests.

Patients were retrospectively categorized based on the occurrence of functional deterioration or ageing events after initiating pirfenidone, regardless of the time elapsed since initiation. In accordance with the inclusion criteria used in clinical trials, all patients were younger than 81 years at the time of starting treatment and had non-advanced IPF, defined as %FVC ≥ 50% and %DLco ≥ 35%. Patients who remained within these criteria (steady state) throughout follow-up were considered the control group. All the others formed the study group, which was divided into three subgroups according to the number of criteria passed. Specifically, patients who progressed beyond one (first criterion passed group, FCPG), two (second criterion passed group, SCPG) or three (third criterion passed group, TCPG) criteria formed the study group and were used for comparisons (Figure 1). The allocation of patients to groups was performed independently from the passed criteria. Thus, FCPG was formed by patients who had passed the age criterion together with those who had passed one functional criterion. In the same way, SCPG was formed by patients who had passed any two criteria. Patients of the SCPG, in the period between passing the first criterion and passing the second one, were included in the FCPG. The same applied for the TCPG. Patients of the SCPG who passed simultaneously two criteria were not included in the FCPG due to the inherent lack of data (not represented in Figure 1). The same applied for the TCPG. Importantly, once a criterion had been passed, the patient was assigned to the study group, regardless of the subsequent trend of respiratory function parameters (not allowed to return to the control group).

### 2.2. Effectiveness Outcomes and Comparisons

Considering that the timing of functional deterioration or ageing events naturally differs between patients, in this study we decided to use the monthly trends of FVC and %DLco as the main outcomes of effectiveness. We decided to consider absolute FVC rather than %FVC because fewer patients had missing data and some of them were obtained with devices with different reference equations. All pulmonary function tests were performed in accordance with ERS/ATS standards, ensuring technical comparability of the measurements [11,12].

To estimate the monthly trend in FVC at steady state or in the time interval between two criteria, we calculate the difference in FVC between the first and the last pulmonary function test performed in that clinical condition and divided it by the number of months elapsed between the two tests. Therefore, the change in FVC between two timepoints was normalized by the number of months between them, yielding an estimated mean monthly rate of change in a specific clinical condition. The same was performed for %DLco. To improve data quality, we excluded from the analysis the respiratory function data for the months corresponding to the progression beyond the criterion (Figure 1). For this reason, the trend in respiratory function parameters of certain phases (e.g., steady state if a patient performed the second pulmonary function test after turning 81 years old) was not used for analysis in some patients.

First, we sought a difference in the monthly trend of respiratory function parameters between the control group and the study group, divided according to the number of criteria passed. Sub-analyses were performed to assess the influence of each specific criterion on the trend. Similarly, we analyzed the trend in patients who had developed an advanced IPF and compared it with that of patients who had passed only a functional criterion together with the age one. Finally, to further explore the intra-individual change in the trend, we compared the trends of each patient before and after the progression beyond a criterion. Sub-analyses were performed even in this case to assess the influence of each specific criterion on the trend.

### 2.3. Therapeutic Management

All patients started pirfenidone at a dose of 267 mg three times daily and reached the full dose of 2403 mg over three weeks. Behavioural modifications and additional medications (i.e., proton pump inhibitors) were recommended to all patients to manage possible adverse events, in particular sunscreen to minimize photosensitivity. Permanent or temporary dose reduction, as well as temporary treatment interruption, were used to manage adverse events. The reduced dose was equal to the highest tolerated one.

### 2.4. Statistical Analysis

Results are reported as mean or median for continuous variables and frequencies with percentage for categorical variables, as appropriate. As dispersion measurement, the standard deviation (SD) and the interquartile range (IQR) were reported. The normality of the distributions was evaluated graphically and formally confirmed using the Shapiro–Wilk test. Comparisons between continuous variables in case of two groups were made using the *t*-test or Mann–Whitney U test, depending on the distribution of the groups. Either one-way ANOVA or the Kruskal–Wallis test was used to compare multiple groups, based on data distribution and variance homogeneity. Post hoc comparisons were performed using Tukey’s HSD (for ANOVA with equal variances), Welch-corrected t-tests with Holm–Bonferroni correction (in case of heteroscedasticity), or Dunn’s test (for Kruskal–Wallis), to control the family-wise error rate. In cases where groups had extremely small sample sizes, an exploratory Kruskal–Wallis analysis was performed only if the smallest group included at least two subjects. Groups containing a single subject were excluded from multiple comparisons tests. For categorical variables, a Chi-squared test was used for comparison between multiple groups, with subsequent use of the Holm–Bonferroni correction. Chi-squared and Fisher’s exact test were used for comparisons between two groups, depending on their size. Paired *t*-test and Wilcoxon test were employed to compare the differences in the trends in those patients who had available data before and after passing a criterion, in accordance with the distribution. Outliers were identified by ROUT method and excluded from the analysis [13]. The results were deemed statistically significant for *p* < 0.05. All statistical evaluations were performed with GraphPad Prism 9.4.1 (GraphPad Software, San Diego, CA, USA).

## 3. Results

### 3.1. Study Population

A total of 182 patients taking pirfenidone were included in the study, but 8 patients were subsequently excluded due to missing data. The mean follow-up time of the remaining 174 was 39.2 months (SD ± 29.7, range 2–152 months). Of these patients, 76 remained within the inclusion criteria used in pivotal clinical trials throughout the follow-up (control group), 72 passed only one criterion (62 %DLco, 9 age and 1 %FVC) and formed the FCPG, while 25 passed two criteria (11 %DLco and %FVC, 13 age and %DLco and 1 age and %FVC) and formed the SCPG. Only 1 patient passed all 3 criteria. Four patients passed simultaneously two criteria, all belonging to the SCPG. The demographic, clinical and functional characteristics of patients are shown in Table 1.

The groups were comparable in terms of demographic characteristics and smoking history. Patients in the control group had a better lung function, which reached statistical significance for %DLco and in part for absolute FVC. Similarly, fewer of them required long-term oxygen therapy.

### 3.2. Comparisons Between Control Group and Study Group

**Figure 2:**
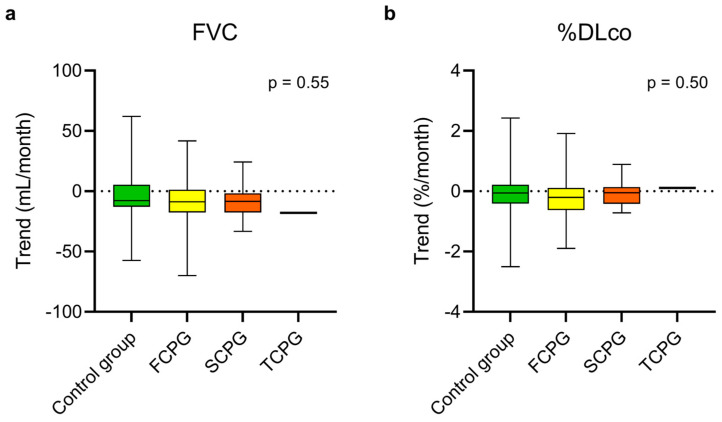
Estimated monthly trends of FVC (**a**) and %DLco (**b**) in the control group and in the three subgroups of the study group during steady state. The vertical lines range from maximum to minimum; the box represents the interquartile range with bar at the median. Kruskal–Wallis test was used for comparisons between the control group and the subgroups of the study group. Data concerning the patient who had passed three criteria are reported for descriptive purposes but were not included in the test. DLco, diffusion lung capacity for carbon monoxide; FVC, forced vital capacity; FCPG, first criterion passed group; SCPG, second criterion passed group; TCPG, third criterion passed group; %DLco, percent predicted DLco value.

**Figure 3:**
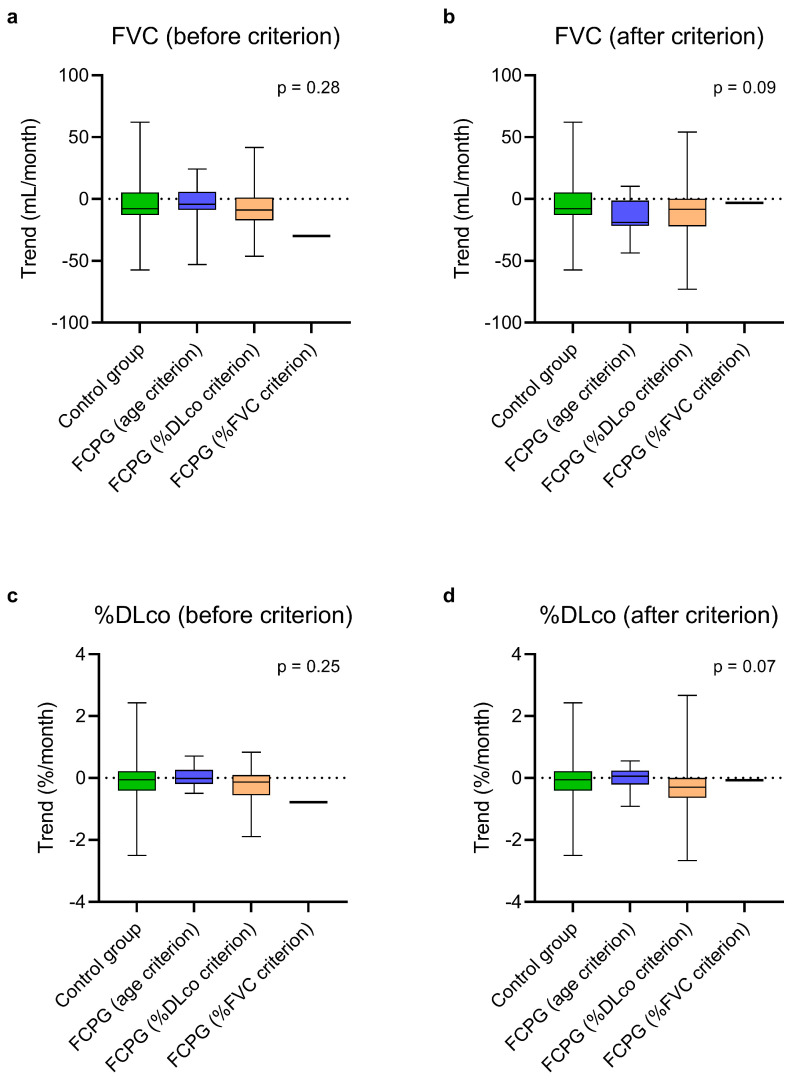
Estimated monthly trends of FVC (**a**,**b**) and %DLco (**c**,**d**) in the control group and in the FCPG, divided into three subgroups based on the passed criterion, at steady state (**a**,**c**) and after passing the first criterion (**b**,**d**). The vertical lines range from maximum to minimum, while the boxes represent the interquartile range with bar at the median. Kruskal–Wallis test was used for comparisons between the control group and the subgroups of the FCPG. Data concerning the patient who had passed the %FVC criterion are reported for descriptive purposes but were not included in the test. DLco, diffusion lung capacity for carbon monoxide; FVC, forced vital capacity; FCPG, first criterion passed group; %DLco, percent predicted DLco value; %FVC, percent predicted FVC value.

**Figure 4:**
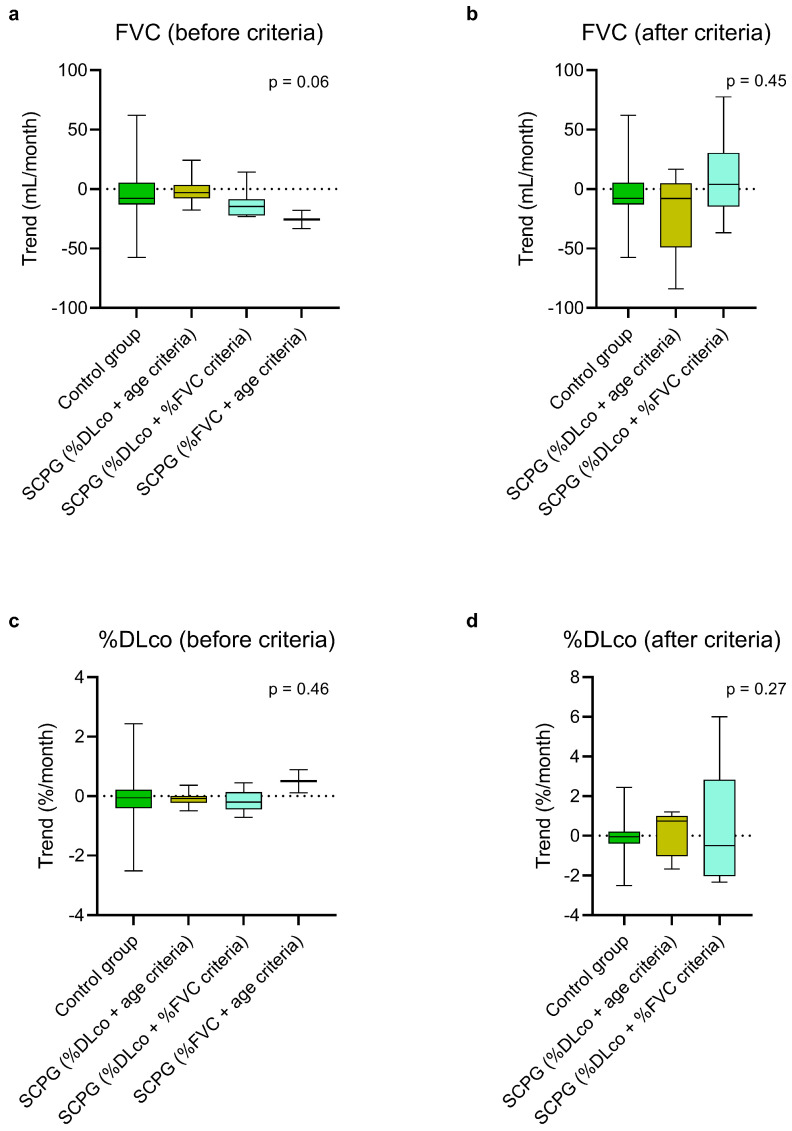
Estimated monthly trends of FVC (**a**,**b**) and %DLco (**c**,**d**) in the control group and in the SCPG, divided into three subgroups based on the passed criteria, at steady state (**a**,**c**) and after passing the criteria (**b**,**d**). The vertical lines range from maximum to minimum, while the boxes represent the interquartile range with bar at the median. Kruskal–Wallis test was used for comparisons between the control group and the subgroups of the SCPG. DLco, diffusion lung capacity for carbon monoxide; FVC, forced vital capacity; SCPG, second criterion passed group; %DLco, percent predicted DLco value; %FVC, percent predicted FVC value.

At steady state, there was no statistically significant difference in the estimated monthly FVC and %DLco trends between the control group, the FCPG, and the SCPG (Figure 2, Table S1). In the same way, no difference emerged comparing the trends of the control group with those of the FCPG (Figure 3, Table S2) and SCPG (Figure 4, Table S3) subgroups.

The estimated monthly trend of FCPG (Figure 3, Table S2) and SCPG (Figure 4, Table S3) subgroups after having passed the criteria was also comparable to that of the control group, concerning both FVC and %DLco.

### 3.3. Comparisons Within the Study Group

**Figure 5:**
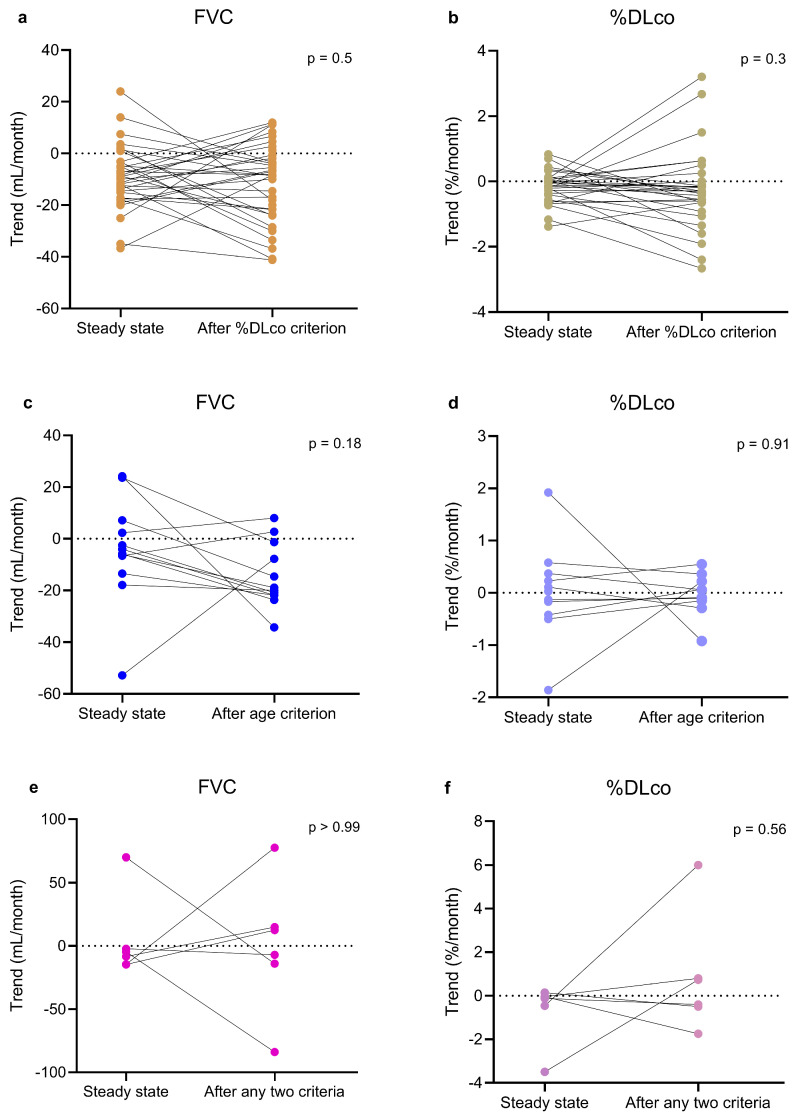
Paired before-after plots illustrating intra-individual estimated monthly trends of FVC (**a**,**c**,**e**) and %DLco (**b**,**d**,**f**) in individual patients before and after passing %DLco criterion (**a**,**b**), age criterion (**c**,**d**), or any two criteria (**e**,**f**). The Figure also includes, in the panels for patients who passed one criterion, data of SCPG and TCPG patients related to the period before the second criterion was passed. Similarly, the Figure also includes, in the panels related to patients who passed two criteria, data of the TCPG patient related to the period before the third criterion was passed. Paired *t*-test and Wilcoxon test were used to compare the patients before and after passing one or two criteria. DLco, pulmonary diffusing capacity of carbon monoxide; FCPG, first criterion passed group; FVC, forced vital capacity; SCPG, second criterion passed group; TCPG, third criterion passed group; %DLco, percent predicted DLco value.

No difference emerged in the estimated monthly trend of both FVC and %DLco when comparing the within-patient trend before and after passing the %DLco or the age criteria (Table 2, Figure 5). Regarding %FVC criterion, no patient had complete data. No difference emerged when considering together all the patients who had passed two criteria as well (Table 2, Figure 5).

## 4. Discussion

The safety and efficacy of pirfenidone in the treatment of mild-to-moderate IPF is well known and several studies have been published to support it [2,3,14,15]. In recent years, some post hoc analyses and real-world studies have also evaluated the safety and efficacy of pirfenidone in patients with more advanced disease (most often defined as %FVC < 50% and/or %DLco < 35%), usually comparing the outcomes of a group of patients with advanced IPF with those of a group of patients with mild-to-moderate disease [4,5,16,17,18,19,20]. Less is known about the role of pirfenidone in elderly patients, and, so far, no study has been conducted to evaluate its effectiveness in patients older than 80 years. In the present study, we evaluated the effectiveness of the drug in a population of patients with IPF who, at the time of prescription, fulfilled the inclusion criteria used by clinical trials, but who progressed beyond one or more of these criteria during their medical history.

In this study, pirfenidone confirmed to remain effective in slowing down the decline of respiratory function even in patients who had progressed beyond the inclusion criteria adopted in clinical trials. Indeed, although numerically less pronounced, the trends of lung function decline after treatment start were comparable to those after progression beyond one criterion. This was observed regardless of the criterion passed. From this point of view, it can be assumed that, at least in our population, patients progressed beyond a criterion simply because they had started pirfenidone with an already more advanced disease and not because their respiratory function declined more rapidly. Indeed, their respiratory function parameters were lower at treatment initiation, in particular %DLco.

When considering the intra-individual respiratory function trend, there was no significant change in the estimated monthly trend before and after progression beyond one or two criteria. In particular, no statistically significant differences in FVC or %DLco were observed after patients had passed the single criterion of age or %DLco, although the trend was often numerically worse. Similarly, analysis of a small group of patients who had passed any two criteria revealed no differences in trend. Even though our data should be interpreted with caution, they support the effectiveness of pirfenidone in patients with progressive disease as well as in the elderly, being in line with previous studies [21]. Progression beyond a criterion should therefore not be a reason to discontinue treatment.

In our population, most patients passed the DLco criterion. In fact, the FCPG and the SCPG already had a lower %DLco than the control group at baseline. Our results highlight that the drug seems to remain effective even in patients with more advanced impairment of this parameter. Patients who progressed and developed advanced IPF according to both %FVC and %DLco often showed stabilization of respiratory function. Although this finding may be partially due to the small sample size and the study design, and in particular the choice not to allow return to the control group after progression beyond a criterion, this supports the ability of pirfenidone to stabilize lung function in patients who develop advanced IPF during treatment. This result is in line with others previously reported in the literature, showing that pirfenidone is able to slow down the decline in patients with a more progressive trend [17,18,22,23]. However, to the best of our knowledge, real-world studies conducted so far have always evaluated the effectiveness of pirfenidone in advanced IPF by analyzing patients starting treatment with already advanced disease, often comparing them to a group of patients with mild-to-moderate disease [17,18,24,25]. Our study, although conducted in a small population, is the first to analyze the trend of respiratory function in patients who develop advanced disease during therapy. On the one hand, our results highlight the variability of progression in IPF, but on the other hand they show that stabilization of the disease can occur even after progression to a very advanced form. From this point of view, our results are in line with those reported by the post hoc analyses of the open-label, long-term extension study RECAP [5].

Even though numerically more pronounced, the decline in FVC and %DLco was not significantly different when patients had become older than 80 years. This result should be interpreted with caution, also because of the small number of patients with available data and possible underpowered analysis, but as far as we know this is the first evidence of the effectiveness of pirfenidone regardless of age. More careful management of adverse events may be needed in elderly patients, even because an increased discontinuation rate has been observed [5,19,26], but advanced age does not seem to limit the effectiveness of the drug.

This study has both limitations and strengths. The first limitation is due to its retrospective and monocentric nature. However, the demographic and functional characteristics of our patients are comparable to those of other studies. The inclusion and exclusion criteria adopted for pulmonary function test increased the strength of our data, but led to a reduction in those analyzable. This decreased the population of some groups, which were sometimes too small to make comparisons, although this was partly balanced by the prolonged period covered. The overall duration of follow-up in this study was indeed longer than in almost all the other studies conducted in patients with advanced IPF. The long time span covered by data collection, however, led to the inclusion of spirometric measurements obtained using different devices. This also represents a limitation, although it was mitigated by using absolute FVC values rather than percentages of the predicted value. As no patient underwent spirometry with different devices on the same day, it was not possible to apply a specific device-related correction. Nevertheless, all pulmonary function tests were performed in accordance with international standards, ensuring the technical comparability of the measurements. Finally, it should be noted that the subgroup analyses were often underpowered and may have been too small to detect either positive or negative therapeutic effects.

## 5. Conclusions

In conclusion, although progression beyond the inclusion criteria used in clinical trials could represent a warning in patients with IPF, data from this study support the effectiveness of pirfenidone in slowing down the decline regardless of functional impairment. Attaining 80 years of age did not appear to limit the effectiveness of pirfenidone, albeit a numerically accelerated decline in respiratory function was noted.

## Figures and Tables

**Table 1:** Characteristics of the groups at the start of treatment and analyzed time.

Characteristic	Control Group (*n* = 76)	Study Group (*n* = 98)		
		One Criterion Passed (*n* = 72)	Two Criteria Passed (*n* = 25)	Three Criteria Passed (*n* = 1)
Age, years				
Mean (SD)	71.9 (6.4)	70.7 (6.3)	73.3 (6.8)	78
Minimum	41	53	54	78
Median	74	71	76	78
Maximum	80	80	80	78
Sex				
Male, *n* (%)	65 (84.4)	59 (81.9)	22 (88)	1 (100)
Smoking history				
Current/former smoker, *n* (%)	56 (73.7)	54 (75)	17 (68)	0
Never smoker, *n* (%)	20 (26.3)	18 (25)	8 (32)	1 (100)
Pack-years	31.9 (21.2)	32.4 (21.5)	22.9 (15.9)	NA
Diagnostic features				
HRCT pattern				
UIP, *n* (%)	56 (73.7)	57 (79.2)	17 (68)	0
UIP probable, *n* (%)	19 (25)	14 (19.4)	8 (32)	1 (100)
Indeterminate for UIP, *n* (%)	1 (1.3)	1 (1.4)	0	0
Lung biopsy, *n* (%)	16 (21)	5 (6.9)	6 (24)	0
Histological pattern				
UIP, *n* (%)	10 (62.5)	3 (60)	2 (33.3)	NA
UIP probable, *n* (%)	4 (25)	1 (20)	3 (50)	NA
Indeterminate for UIP, *n* (%)	2 (12.5)	1 (20)	1 (16.7)	NA
Functional parameters				
FVC, l	2.7 (0.6)	2.7 (0.7)	2.4 (0.4) **°	2.9
FVC, % of predicted value	86.5 (18.3)	82.3 (16.9)	75 (14.2)	85
DLco, % of predicted value	56 (14.5)	49.2 (11.8) **	48.3 (11.2) *	63
Home oxygen therapy, *n* (%)	4 (5.3)	16 (22.2) **	6 (24) *	0
BMI, kg/m^2^	27.6 (4)	29.5 (4.5)	32.3 (4)	28.2
Prognostic tools				
GAP index, points	3.6 (1)	3.6 (0.9)	4.2 (1.1)	2
Analyzed time				
Steady state, months	29.8 (30.3)	21.3 (23.2)	23.1 (21.4)	28
After first criterion, months	NA	14.3 (16.9)	10.1 (12.4)	24
After second criterion, months	NA	NA	4.2 (8.3)	0
After third criterion, months	NA	NA	NA	0

Results are reported as mean and standard deviation or as number of patients and percentage, as appropriate. Data concerning the patient who had passed three criteria are reported for descriptive purposes but were not included in the analyses. BMI, body mass index; DLco, diffusion lung capacity for carbon monoxide; FVC, forced vital capacity; GAP, gender-age-physiology; HRCT, high resolution computer tomography; LTOT, long-term oxygen therapy; NA, not applicable; SD, standard deviation; UIP, usual interstitial pneumonia; *, *p* < 0.05 versus control group; **, *p* < 0.01 versus control group; °, *p* < 0.05 versus one criterion passed group.

**Table 2:** Comparison of the within-patient estimated monthly trend of respiratory function parameters before and after passing the criteria.

Parameter	%DLco Criterion (*n* = 73)	Age Criterion (*n* = 18)	%FVC Criterion (*n* = 2)	Any Two Criteria (*n* = 26)
Steady state				
FVC, mL/month				
N. of patients with data	33	12	0	6
Mean (SD)	−9.02 (18.03)	−4.32 (20.02)		4.25 (32.6)
Median (IQR)	−9.04 (−17.17–−3.17)	−4.91 (−11.75–5.93)		−6.5 (−14.6–15.84)
%DLco, %/month				
N. of patients with data	30	10	0	6
Mean (SD)	−0.16 (0.48)	0.01 (0.9)		−0.67 (1.4)
Median (IQR)	−0.12 (−0.45–0.09)	−0.01 (−0.44–0.42)		−0.08 (−1.22–0)
After passing a criterion				
FVC, mL/month				
N. of patients with data	33	12	0	NA
Mean (SD)	−11.24 (15.16)	−14.52 (12.41)		
Median (IQR)	−8.33 (−22.66–−1.2)	−19.45 (−21.82–−2.93)		
*p*-value	0.497	0.178		
%DLco, %/month				
N. of patients with data	30	10	0	NA
Mean (SD)	−0.27 (1.23)	−0.03 (0.4)		
Median (IQR)	−0.31 (−0.71–0.06)	−0.01 (−0.18–0.25)		
*p*-value	0.301	0.913		
After passing two criteria				
FVC, ml/month				
N. of patients with data	NA	NA	NA	6
Mean (SD)				0 (52.32)
Median (IQR)				2.75 (−31.5–30.63)
*p*-value				>0.999
%DLco, %/month				
N. of patients with data	NA	NA	NA	6
Mean (SD)				0.81 (2.71)
Median (IQR)				0.16 (−0.81–2.1)
*p*-value				0.562

Results are reported as mean with standard deviation and as median with interquartile range. The table also includes, in the columns for patients who passed one criterion, data of SCPG and TCPG patients related to the period before the second criterion was passed. Similarly, the Table also includes, in the column related to patients who passed two criteria, data of the TCPG patient related to the period before the third criterion was passed. Paired *t*-test and Wilcoxon test were used to compare the patients before and after passing one or two criteria. DLco, pulmonary diffusing capacity of carbon monoxide; FVC, forced vital capacity; IQR, interquartile range; NA, not applicable; SCPG, second criterion passed group; TCPG, third criterion passed group; SD, standard deviation; %FVC, percent predicted FVC value; %DLco, percent predicted DLco value.

## Data Availability

The data presented in this study are available on reasonable request from the corresponding author (the data are not publicly available due to privacy and ethical restrictions).

## Supplementary Materials

The following supporting information can be downloaded at https://www.mdpi.com/article/10.3390/biomedicines13112809/s1, Table S1: Monthly trends of respiratory function parameters in the control group and in the study group during steady state; Table S2: Monthly trends of respiratory function parameters in the control group and in the first criterion passed group; Table S3: Monthly trends of respiratory function parameters in the control group and in the second criterion passed group.

## Abbreviations

The following abbreviations are used in this manuscript:

ATS	American Thoracic Society
BMI	Body mass index
DLco	Diffusion capacity of the lung for carbon monoxide
ERS	European Respiratory Society
FCPG	First criterion passed group
FVC	Forced vital capacity
GAP	Gender-age-physiology
HRCT	High resolution computer tomography
IPF	Idiopathic pulmonary fibrosis
IQR	Interquartile range
LTOT	Long-term oxygen therapy
NA	Not applicable
PFTs	Pulmonary function tests
SCPG	Second criterion passed group
SD	Standard deviation
TCPG	Third criterion passed group
UIP	Usual interstitial pneumonia
%DLco	Percentage of predicted DLco value
%FVC	Percentage of predicted FVC value
