# Supplementary material for: Maintenance of Effectiveness of Pirfenidone in Elderly Patients with Progressive Functional Impairment: A Real-World Retrospective Study in IPF
Source: Biomedicines. 2025 Nov 18;13(11):2809. doi: 10.3390/biomedicines13112809 (PMC12650561; doi:10.3390/biomedicines13112809)
Supplement: Supplementary file 1 [file biomedicines-13-02809-s001.zip › biomedicines-3930883-supplementary.pdf]

Maintenance of effectiveness of pirfenidone in elderly patients with progressive functional impairment: a real-world retrospective study in IPF

Stefano Levra[1], Cecilia Rivero[1], Fabiana Giannoccaro[2], Giuseppe Guida[1,2], Francesca Bertolini[1], Vitina Carriero[1], Elisa Arrigo[1], Maurizio Balbi[3], Carlo Albera[4,5], Fabio L. M. Ricciardolo[1,2]

Results

Supplementary Table S1. Monthly trends of respiratory function parameters in the control group and in the study group during steady state.

Characteristic	Control group (n=76)	Study group (n=98)			p-value
		One criterion passed (n=72)	Two criteria passed (n=25)	Three criteria passed (n=1)	
FVC, mL/month					0.5456
N. of patients with data	72	56	19	1	
Mean (SD)	-2.87 (23.02)	-9.1 (21.11)	-7.79 (13.79)	-17.86	
Median (IQR)	-7.74 (-12.85 – 5.34)	-8.78 (-17.61 – 1.32)	-8.38 (-17.6 – -1.77)	-17.86	
%DLco, %/month					0.4954
N. of patients with data	71	55	19	1	
Mean (SD)	-0.07 (0.83)	-0.23 (0.69)	-0.08 (0.38)	0.11	
Median (IQR)	-0.06 (-0.41 – 0.22)	-0.2 (-0.62 – 0.11)	-0.05 (-0.42 – 0.14)	0.11	

Results are reported as mean with standard deviation and as median with interquartile range. Kruskal-Wallis test was used for comparisons between the control group and the subgroups of the study group. Data concerning the patient who had passed three criteria are reported for descriptive purposes but were not included in the test. DLco, diffusion lung capacity for carbon monoxide; FVC, forced vital capacity; IQR, interquartile range; SD, standard deviation; %DLco, percent predicted DLco value.

Supplementary Table S2. Monthly trends of respiratory function parameters in the control group and in the first criterion passed group.

Parameter	Control group (n=76)	First criterion passed group			p-value
		%DLco criterion (n=73)	Age criterion (n=18)	%FVC criterion (n=2)	
**Steady state**					
FVC, mL/month					0.2846
N. of patients with data	72	54	16	1	
Mean (SD)	-2.87 (23)	-7.17 (15.97)	-2.96 (18.32)	-30	
Median (IQR)	-7.74 (-12.85 – 5.34)	-8.87 (-17.28 – 1.07)	-4.31 (-8.68 – 5.93)	-30	
%DLco, %/month					0.2521
N. of patients with data	71	55	14	1	
Mean (SD)	-0.07 (0.83)	-0.25 (0.57)	0.03 (0.35)	-0.78	
Median (IQR)	-0.06 (-0.41 – 0.22)	-0.14 (-0.56 – 0.1)	-0.02 (-0.2 – 0.26)	-0.78	
**After passing the criterion**					
FVC, mL/month					0.0908
N. of patients with data	NA	50	15	1	
Mean (SD)		-10.07 (19.98)	-14.63 (15.04)	-3.1	
Median (IQR)		-8.33 (-22.16 – 0.31)	-18.89 (-21.82 – -1.32)	-3.1	
%DLco, %/month					0.0651
N. of patients with data	NA	44	13	1	
Mean (SD)		-0.34 (0.95)	-0.03 (0.38)	-0.07	
Median (IQR)		-0.29 (-0.64 – 0)	0.06 (-0.22 – 0.23)	-0.07	

Results are reported as mean with standard deviation and as median with interquartile range. The Table includes data of the patients of the SCPG and the TCPG related to the period before passing

the second criterion. Kruskal-Wallis test was used for comparisons between the control group and the subgroups of the first criterion passed group. Data concerning the patient who had passed the %FVC criterion are reported for descriptive purposes but were not included in the test. DLco, diffusion lung capacity for carbon monoxide; FVC, forced vital capacity; IQR, interquartile range; NA, not applicable; SCPG, second criterion passed group; SD, standard deviation; TCPG, third criterion passed group; %FVC, percent predicted FVC value; %DLco, percent predicted DLco value.

Supplementary Table S3. Monthly trends of respiratory function parameters in the control group and in the second criterion passed group.

Parameter	Control group (n=76)	Second criterion passed group			p-value
		%DLco and %FVC criteria (n=11)	%DLCO and age criteria (n=13)	%FVC and age criteria (n=2)	
**Steady state**					
FVC, mL/month					0.0589
N. of patients with data	72	8	10	2	
Mean (SD)	-2.87 (23)	-12.31 (12.18)	-1.61 (11.49)	-25.6 (10.94)	
Median (IQR)	-7.74 (-12.85 – 5.34)	-14.65 (-22.33 – -8.5)	-3.11 (-7.92 – 3.52)	-25.6 (-33.33 – -17.86)	
%DLco, %/month					0.4622
N. of patients with data	71	8	10	2	
Mean (SD)	-0.07 (0.83)	-0.16 (0.39)	-0.1 (0.24)	0.5 (0.55)	
Median (IQR)	-0.06 (-0.41 – 0.22)	-0.2 (-0.45 – 0.14)	-0.08 (-0.23 – 0.01)	0.5 (0.11 – 0.89)	
**After passing two criteria**					
FVC, mL/month					0.4516
N. of patients with data	NA	6	5	0	
Mean (SD)		9.4 (38.15)	-19.27 (38.04)		
Median (IQR)		3.97 (-14.71 – 30.63)	-8 (-49 – 4.83)		
%DLco, %/month					0.2742
N. of patients with data	NA	5	5	0	
Mean (SD)		0.21 (3.34)	0.13 (1.17)		
Median (IQR)		-0.5 (-2.04 – 2.82)	0.73 (-1.03 – 1)		

Results are reported as mean with standard deviation and as median with interquartile range. The Table includes data of the patient of the TCPG related to the period before passing the third criterion. Kruskal-Wallis test was used for comparisons between the control group and the three subgroups of the second criterion passed group. DLco, diffusion lung capacity for carbon monoxide; FVC, forced vital capacity; IQR, interquartile range; NA, not applicable; SD, standard deviation; TCPG, third criterion passed group; %FVC, percent predicted FVC value; %DLco, percent predicted DLco value.
